# An Upregulation in the Expression of Vanilloid Transient Potential Channels 2 Enhances Hypotonicity-Induced Cytosolic Ca^2+^ Rise in Human Induced Pluripotent Stem Cell Model of Hutchinson Gillford Progeria

**DOI:** 10.1371/journal.pone.0087273

**Published:** 2014-01-27

**Authors:** Chun-Yin Lo, Yung-Wui Tjong, Jenny Chung-Yee Ho, Chung-Wah Siu, Sin-Ying Cheung, Nelson L. Tang, Shan Yu, Hung-Fat Tse, Xiaoqiang Yao

**Affiliations:** 1 School of Biomedical Sciences, The Chinese University of Hong Kong, Hong Kong, China; 2 Division of Cardiology, Department of Medicine, The University of Hong Kong, Queen Mary Hospital, Hong Kong, China; 3 Department of Chemical Pathology, The Chinese University of Hong Kong, Hong Kong, China; 4 School of Chinese Medicine, Hong Kong Baptist University, Hong Kong, China; Indiana University School of Medicine, United States of America

## Abstract

Hutchinson-Gillford Progeria Syndrome (HGPS) is a fatal genetic disorder characterized by premature aging in multiple organs including the skin, musculoskeletal and cardiovascular systems. It is believed that an increased mechanosensitivity of HGPS cells is a causative factor for vascular cell death and vascular diseases in HGPS patients. However, the exact mechanism is unknown. Transient receptor potential (TRP) channels are cationic channels that can act as cellular sensors for mechanical stimuli. The aim of this present study was to examine the expression and functional role of TRP channels in human induced pluripotent stem cell-derived endothelial cells (iPSC-ECs) from the patients with HGPS. The mRNA and protein expression of TRP channels in HGPS and control (IMR90) iPSC-ECs were examined by semi-quantitative RT-PCRs and immunoblots, respectively. Hypotonicity-induced cytosolic Ca^2+^ ([Ca^2+^]_i_) rise in iPSC-ECs was measured by confocal microscopy. RT-PCRs and immunoblots showed higher expressional levels of TRPV2 in iPSC-ECs from HGPS patients than those from normal individuals. In functional studies, hypotonicity induced a transient [Ca^2+^]_i_ rise in iPSC-ECs from normal individuals but a sustained [Ca^2+^]_i_ elevation in iPSC-ECs from HGPS patients. A nonselective TRPV inhibitor, ruthenium red (RuR, 20 µM), and a specific TRPV2 channel inhibitor, tranilast (100 µM), abolished the sustained phase of hypotonicity-induced [Ca^2+^]_i_ rise in iPSC-ECs from HGPS patients, and also markedly attenuated the transient phase of the [Ca^2+^]_i_ rise in these cells. Importantly, a short 10 min hypotonicity treatment caused a substantial increase in caspase 8 activity in iPSC-ECs from HGPS patients but not in cells from normal individuals. Tranilast could also inhibit the hypotonicity-induced increase in caspase 8 activity. Taken together, our data suggest that an up-regulation in TRPV2 expression causes a sustained [Ca^2+^]_i_ elevation in HGPS-iPSC-ECs under hypotonicity, consequently resulting in apoptotic cell death. This mechanism may contribute to the pathogenesis of vascular diseases in HGPS patients.

## Introduction

Hutchinson-Gillford progeria syndrome (HGPS) is a fatal genetic disorder characterized by premature aging in multiple organs including skin, musculoskeletal and cardiovascular systems [1], [2]. HGPS patients suffer from early severe cardiovascular diseases which are characterized by progressive atherosclerosis [3], [4], adventitial fibrosis and left ventricular hypertrophy [5]. They typically die from myocardial infarction or ischemic attack at the average age of 13 [6]. HGPS belongs to laminiopathies associated with point mutations in nuclear A/C lamin gene (LMNA), which results in the production of a truncated lamin A protein known as progerin. Lack of normal lamin A and accumulation of progerin result in abnormal nuclear envelope shape and chromatin architectures [7], [8], and causes disruption of cell division [9], [10]. A recent study showed that cytosolic Ca^2+^ may cause a conformational change in progerin, affecting its posttranslational processing, which may be crucial for disease pathogenesis [11]. However, the mechanisms of premature atherosclerosis and death in HGPS patients remain obscure.

Studies have showed that HGPS cells display an increase in mechanosensitivity [9], [10], which may be associated with a decrease in viability and an increase in apoptosis under repetitive mechanical strain [9]. Vascular cells, including vascular smooth muscle cells and endothelial cells, are the primary targets of progerin accumulation [10]. These cells are constantly exposed to fluid shear stress and mechanical strain in the vessel wall [9]. Moreover, it has been suggested that an increase in mechanical sensitivity of vascular cells from HGPS patients may impair cell cycle activation, which contributes to necrotic and apoptotic vascular cell death, leading to severe vascular diseases including atherosclerosis [9]. Although an increase in mechanosensitivity of HGPS vascular cells appears to play a critical role in vascular cell death and cardiovascular diseases, there are still no reports on “mechanosensors” that serve to monitor mechanical stimuli in HGPS vascular cells.

Transient receptor potential (TRP) channels are nonselective cation channels that comprise of six subfamilies: TRPC (canonical), TRPM (melastatin), TRPML (mucolipin), TRPP (polycystin), TRPA (ankyrin) and TRPV (vanilloid) [12]. These channels function to perceive and respond to various environmental stimuli, such as thermal, heat, pain, pH, mechanical and osmotic stress [12], [13]. TRP channels have been implicated in several cardiovascular diseases including atherosclerosis [14], hypertension [15], [16], vascular remodeling, and cardiac hypertrophy [17]. Most TRP channels are Ca^2+^-permeable. Mechano-activation of these TRP channels results in Ca^2+^ influx. Excessive Ca^2+^ influx or Ca^2+^ overload are known to contribute to apoptotic and necrotic vascular cell death [14], [17]–[19].

Our recent studies showed that human induced pluripotent stem cell (iPSC) could be derived from normal subjects as well as from patients with HGPS [20]–[22]. Moreover, iPSC-derived vascular cells from HGPS patients are a good model for studying the correlation between premature senescence phenotypes and vascular ageing [21]. The aim of this present study was to explore the possible role of TRP channels in mechanosensation in iPSC-derived endothelial cells (iPSC-ECs) from HGPS patients.

## Results

### Hypotonicity-induced [Ca^2+^]_i_ rise in iPSC-ECs from HGPS patients and normal individuals

**Figure 1:**
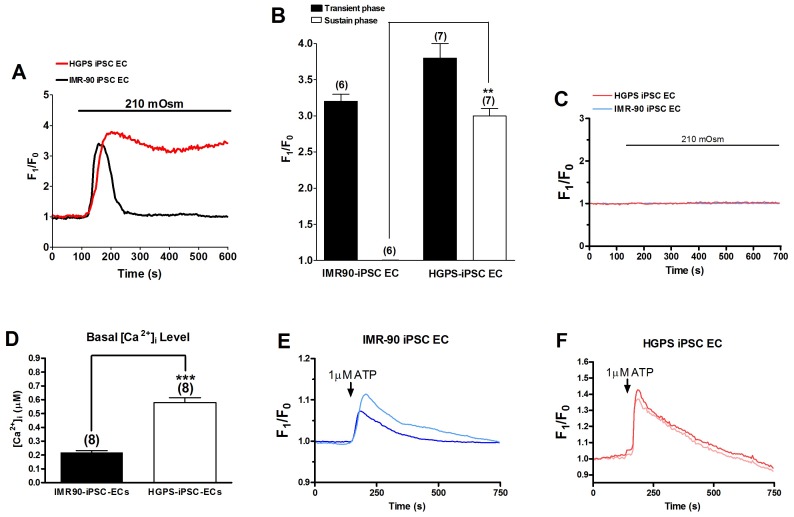
Effect of hypotonicity and ATP on [Ca^2+^]_i_ in IMR90-iPSC-ECs and HGPS-iPSC-ECs. (A and B), Representative traces (A) and data summary (B) showing the effect of hypotonicity (210 mOsm) on [Ca^2+^]_i_ (fluorescence ratio F1/F0) in IMR90-iPSC-ECs and HGPS-iPSC-ECs bathed in isotonic solution. n  =  6-7 experiments. (C), Representative traces showing the effect of hypotonic solution (210 mOsm) on [Ca^2+^]_i_ in cells bathed in Ca^2+^-free isotonic saline. n  =  8 experiments. D. Basal [Ca^2+^]_i_ level in IMR90-iPSC-ECs and HGPS-iPSC-ECs as determined by Fura-2 dye. n  =  8. ^**^ *p*<0.01 unpaired *t*-test compared with the sustained [Ca^2+^]_i_ level in IMR90-iPSC-EC group in B or compared with basal [Ca^2+^]_i_ level in D. (E and F), Representative traces showing the effect of ATP (1 µM) on [Ca^2+^]_i_ in cells bathed in normal physiological saline. Representative from 3 experiments.

We used a single wavelength dye Fluo-4 to measure [Ca^2+^]_i_ change. Figure 1A and 1B show the effect of hypotonicity on [Ca^2+^]_i_ in the iPSC-ECs derived from normal individuals (IMR90-iPSC-ECs) and HGPS patients (HGPS-iPSC-ECs), respectively. Upon perfusion with hypotonic solution (210 mOsm), [Ca^2+^]_i_ increased rapidly and peaked within 54±7 sec in IMR90-iPSC-ECs and 67±4 sec in HGPS-iPSC-ECs. Subsequently, elevated [Ca^2+^]_i_ gradually reverted back to its resting level in IMR90-iPSC-ECs with F1/F0 value of 1.0±0.1 (n  =  6). In contrast, [Ca^2+^]_i_ elevation in HGPS-iPSC-ECs sustained with the F1/F0 value maintained at ∼3.0 over 10 min (n  =  7) (Figure 1A and B). In the absence of extracellular Ca^2+^, hypotonicity failed to induce [Ca^2+^]_i_ rise in both cell types (Figure 1C), suggesting that the hypotonicity-induced [Ca^2+^]_i_ rise was mostly due to Ca^2+^ influx but not intracellular Ca^2+^ store release. Basal [Ca^2+^]_i_ level was measured using a dual wavelength ratiometric dye Fura-2. The basal [Ca^2+^]_i_ level was found to be significantly higher in HGPS-iPSC-ECs than in IMR90-iPSC-ECs (Figure 1D). [Ca^2+^]_i_ response to extracellular ATP challenge was also examined. Unlike the responses to hypotonicity, ATP application (1 µM) only induced transient [Ca^2+^]_i_ rise in both cell types. The magnitude of transient [Ca^2+^]_i_ response to ATP was slightly higher in HGPS-iPSC-ECs than that in IMR90-iPSC-ECs (Figure 1E and F). However, because it is well documented that ATP-induced [Ca^2+^]_i_ transients in endothelial cells were mostly due to Ca^2+^ release from IP_3_-sensitive intracellular Ca^2+^ stores [23] but unrelated to mechanosensitive Ca^2+^ influx, no further studies were carried out.

### An increase in TRPV2 expression in HGPS-iPSC-ECs

**Figure 2:**
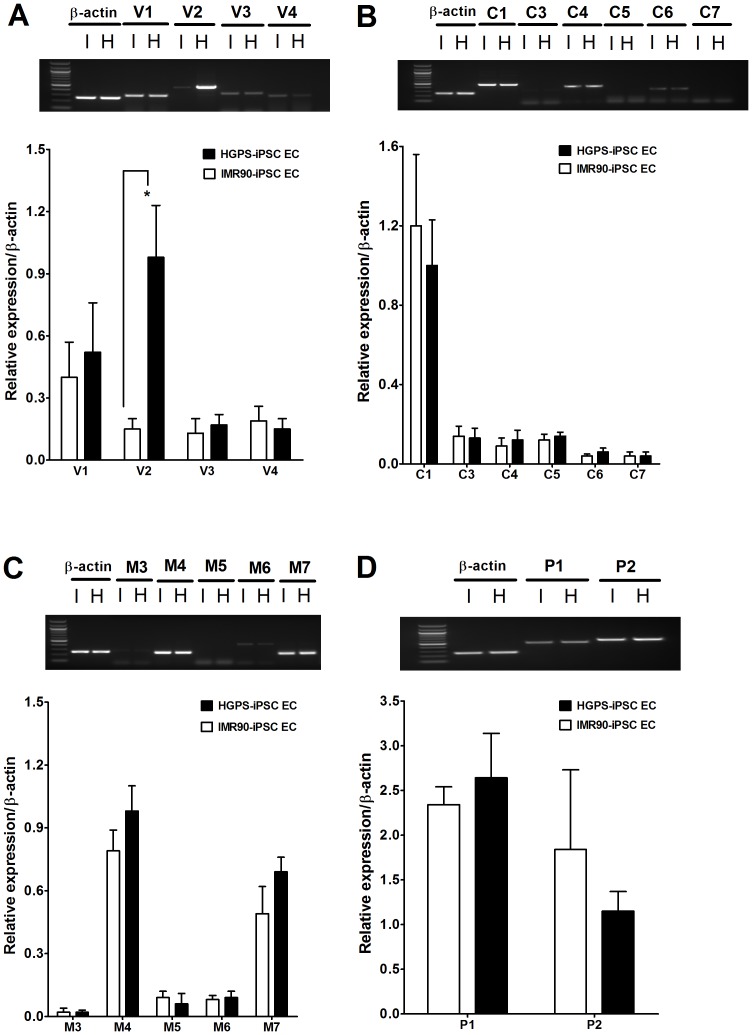
Expression of TRP channel transcripts in IMR90-iPSC-ECs and HGPS-iPSC-ECs. Shown were the expressional levels of transcripts for TRPV (A), TRPC (B), TRPM (C) and TRPP (D) in IMR90-iPSC-ECs (I) and HGPS-iPSC-ECs (H). n  =  4 independent experiments. ^*^ *p*<0.05 unpaired *t*-test compared with IMR90-iPSC-ECs.

**Figure 3:**
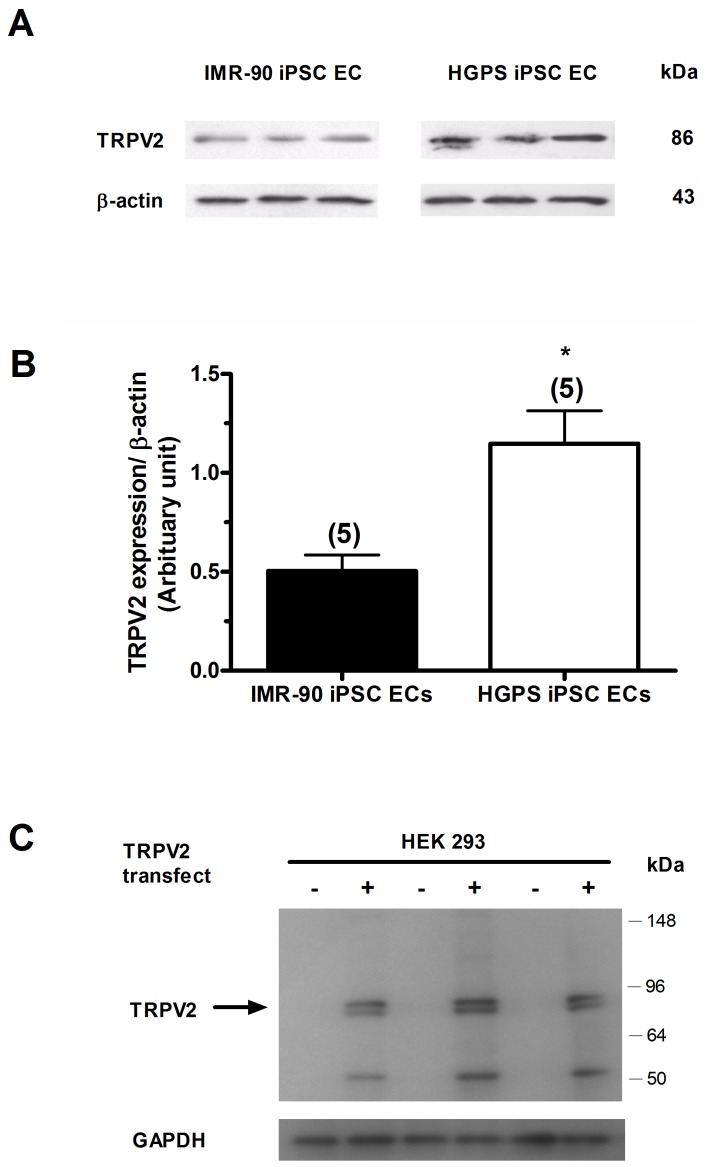
Expression of TRPV2 proteins in IMR90-iPSC-ECs and HGPS-iPSC-ECs. (A and B), representative images (A) and data summary (B) of TRPV2 protein expression in IMR90- and HGPS-iPSC-ECs. n  =  5 experiments. ^*^ *p*<0.05 unpaired *t*-test compared with IMR90-iPSC-EC. (C), Representative immunoblot images showing that the TRPV2-antibody recognized the targeted bands in TRPV2-overexpressing HEK293 cells (+) but not in non-transfected HEK293 cells (-). n  =  3 experiments.

mRNA levels of TRPV1/2/3/4, TRPC1/3/4/5/6/7, TRPM3/4/5/6/7 and TRPP1/2 channels in IMR90-iPSC-ECs and HGPS-iPSC-ECs were determined by semi-quantitative RT-PCRs. Results showed detectable expression of all TRP isoform mRNAs (n  =  4 experiments for each group) in both cell types (Figure 2A to 2D). In particular, the mRNA level of TRPV2 channels was significantly higher in HGPS-iPSC-ECs compared to IMR90-iPSC-ECs (Figure 2A). Furthermore, the protein expression of TRPV2 channels in IMR90-iPSC-ECs and HGPS-iPSC-ECs was determined by immunoblots (Figure 3A). The expression level of TRPV2 proteins was 2.3 times higher in HGPS-iPSC-ECs (n  =  5, *p*<0.05) than in IMR90-iPSC-ECs (n  =  5) (Figure 3B). The specificity of anti-TRPV2 antibody was verified. The TRPV2 antibody could recognize expected TRPV2 bands in TRPV2-overexpressing HEK293 cells (Figure 3C), similar to previous reports by others [24]. No bands were observed in non-transfected HEK293 cells (Figure 3C).

### The effects of TRPV2 inhibitors on hypotonicity-induced [Ca^2+^]_i_ rise

**Figure 4:**
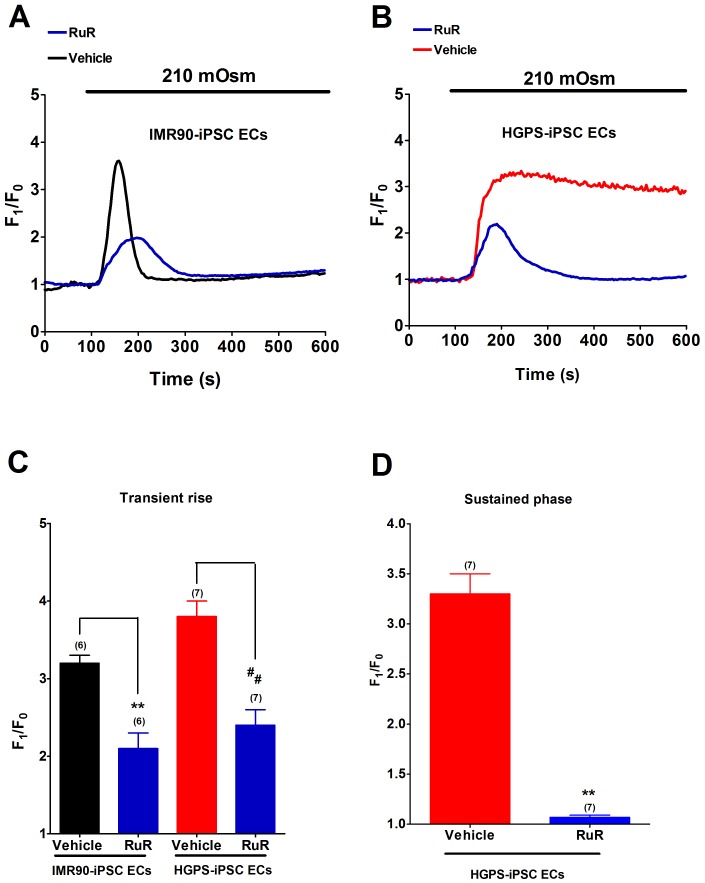
Effect of ruthenium red (RuR) on hypotonicity-induced [Ca^2+^]_i_ rise. (A-B), Representative traces showing the effect of RuR (20 µM) on hypotonicity-induced [Ca^2+^]_i_ rise in IMR90-iPSC-ECs (A) and HGPS-iPSC-ECs (B). (C and D), Summarized data showing the effect of RuR on transient phase (C) and sustained phase (D) of hypotonicity-induced [Ca^2+^]_i_ rise. n  =  6–7 independent experiments, 5–10 cells per experiment. ^**^ *p*<0.01 or ^##^ *p*<0.01 unpaired *t*-test compared with corresponding vehicle control.

**Figure 5:**
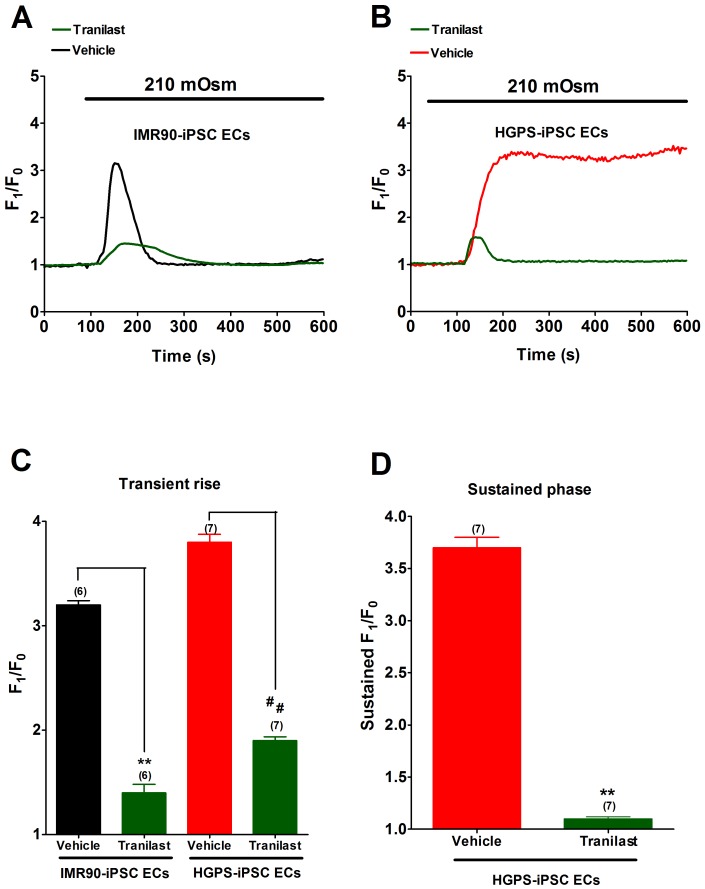
Effect of tranilast on hypotonicity-induced [Ca^2+^]_i_ rise. (A-B), Representative traces showing the effect of tranilast (100 µM) on hypotonicity-induced [Ca^2+^]_i_ rise in IMR90-iPSC-ECs (A) and HGPS-iPSC-ECs (B). (C and D), Summarized data showing the effect of tranilast on transient phase (C) and sustained phase (D) of hypotonicity-induced [Ca^2+^]_i_ rise. n  =  6–7 independent experiments, 5–10 cells per experiment. ^**^ *p*<0.01 unpaired *t*-test compared with vehicle control.

TRPV2 has been known to be a mechanosensitive channel [25], [26]. To determine whether TRPV2 was involved in hypotonicity-induced [Ca^2+^]_i_ influx, a nonselective TRPV inhibitor ruthenium red (RuR) and a TRPV2-specific inhibitor tranilast were used. RuR (20 µM) completely abolished the sustained phase of hypotonicity-induced [Ca^2+^]_i_ rise in HGPS-iPSC-ECs (Figure 4D). The agent also substantially inhibited the transient phase of hypotonicity-induced [Ca^2+^]_i_ rise by about 34% and 36% in IMR90-iPSC-ECs and HGPS-iPSC-ECs, respectively (Figure 4C). Similarly, tranilast (100 µM) abolished the sustained phase of hypotonicity-induced [Ca^2+^]_i_ rise in HGPS-iPS-ECs (Figure 5D), and also substantially inhibited the transient phase of hypotonicity-induced [Ca^2+^]_i_ rise by 50% (n  =  6, *p*<0.01) and 56% (n  =  6, *p*<0.01) in IMR90-iPSC-ECs and HGPS-iPSC-ECs, respectively (Figure 5C).

### The effects of TRPV2 inhibitors on hypotonic-induced caspase 8 activation

**Figure 6:**
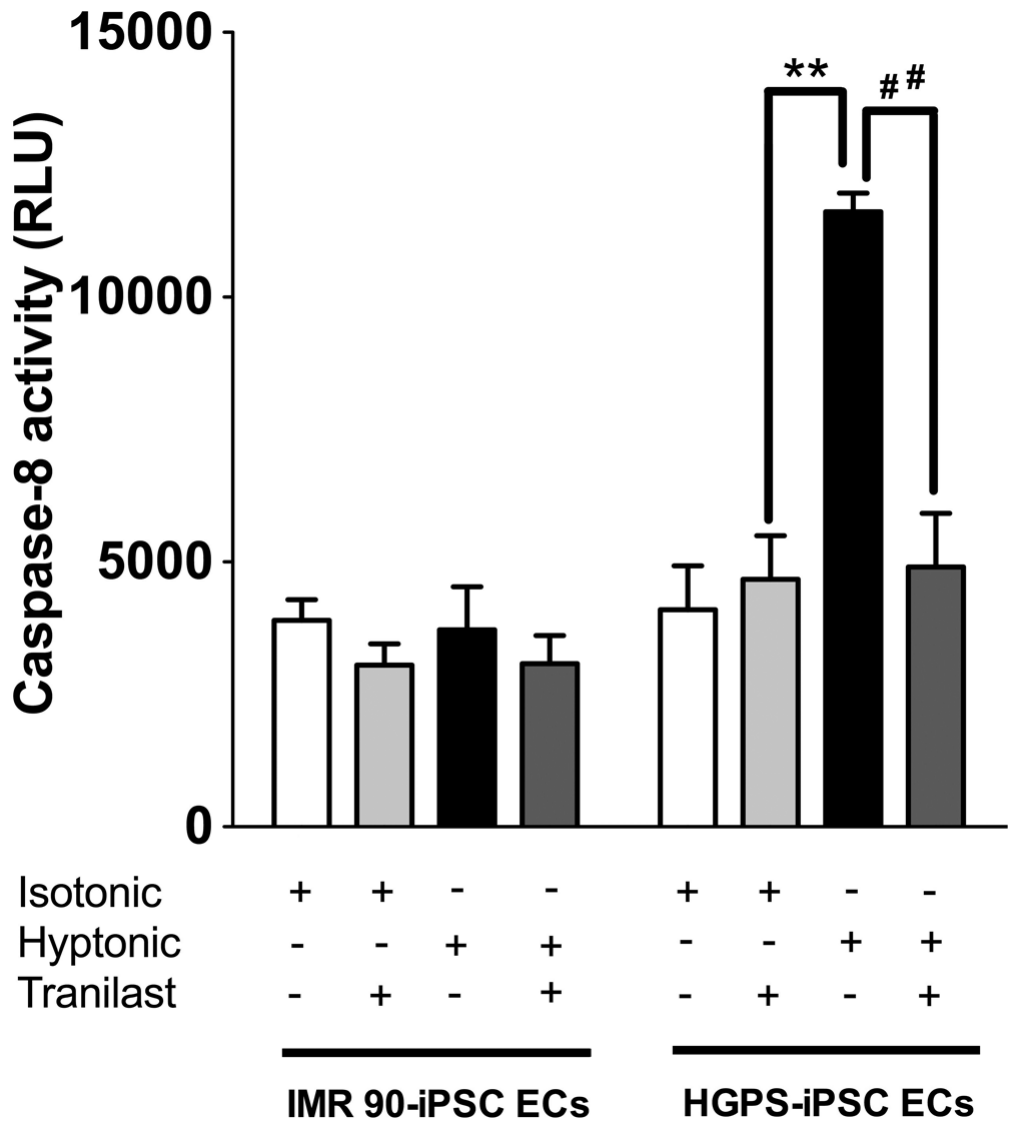
Effect of tranilast on hypotonicity-stimulated caspase-8 activity. Data summary showing the inhibitory effect of tranilast on hypotonicity-induced activation of caspase-8 in HGPS-iPSC-ECs. n  =  6 independent experiments for each group. ^**^ *p*<0.01 unpaired *t*-test compared with isotonicity. ^##^ *p*<0.01 unpaired t-test compared with hypotonicity without tranilast.

The effect of hypotonicity on cellular apoptosis was determined by measuring caspase 8 activity, which has been reported as a hallmark of apoptotic cell death [27]. Hypotonicity treatment for 10 min significantly increased caspase 8 activity in HGPS-iPSC-ECs but not in IMR90-iPSC-ECs (Figure 6). The hypotonicity-induced caspase 8 activation in the HGPS-iPSC-ECs was abolished by 100 µM tranilast treatment (Figure 6).

## Discussion

Major findings of the present study are as follows: 1) iPSC-ECs from HGPS patients expressed much higher levels of TRPV2 mRNAs and proteins compared to iPSC-ECs from normal individuals. 2) Hypotonicity induced a transient [Ca^2+^]_i_ rise in iPSC-ECs from normal individuals, but evoked a sustained [Ca^2+^]_i_ elevation in iPSC-ECs from HGPS patients. 3) The sustained phase of hypotonicity-induced [Ca^2+^]_i_ rise was completely abrogated by tranilast or RuR in iPSC-ECs from HGPS patients. Furthermore, the transient phase of hypotonicity-induced [Ca^2+^]_i_ rise was partially inhibited by RuR or tranilast. 4) Hypotonicity treatment for 10 min caused substantial increase in caspase 8 activity in iPSC-ECs from HGPS patients but not in iPSC-ECs from normal individuals. Tranilast could inhibit the hypotonicity-induced increase in caspase 8 activity. Taken together, our data strongly suggested that TRPV2 was a major mechanosensitive channel that contributed to hypotonicity-induced [Ca^2+^]_i_ rise in iPSC-ECs. More importantly, this channel was exclusively responsible for sustained Ca^2+^ influx under hypotonicity and contributed to apoptotic cell death in HGPS-iPSC-ECs.

It has been well documented that most TRP channels are Ca^2+^-permeable and many of them, including TRPC1, TRPC5, TRPV2, TRPV4, TRPM3, TRRM7 and TRPP2, can be activated by hypotonicity-induced cell swelling [28]. Thus, we compared the expression profile of TRP channel mRNAs in iPSC-ECs from HGPS patients with that from normal individuals. iPSC-ECs from both subjects expressed multiple hypotonicity-activated TRP channels, including TRPC1, TRPV2, TRPV4, TRPM7 and TRPP2. However, while expression levels of other TRP channels remained comparable, TRPV2 displayed a higher expression level in HGPS-iPSC-ECs than that in IMR90-iPSC-ECs. Such observation was confirmed by immunoblots. This prompted us to determine whether TRPV2 accounted for the unique phenomenon of sustained [Ca^2+^]_i_ elevation in HGPS-iPSC-ECs. The results showed that both RuR and tranilast could abolish the sustained phase of hypotonicity-induced [Ca^2+^]_i_ rise, suggesting that TRPV2 was the sole component in mediating sustained Ca^2+^ entry in response to hypotonicity in these cells. Both RuR and tranilast could also substantially reduce the transient phase of hypotonicity-induced [Ca^2+^]_i_ rise by 50–60% in both cell types, indicating that TRPV2 also contributed significantly to the transient phase of [Ca^2+^]_i_ rise. The residual component (∼40%) in the transient phase of [Ca^2+^]_i_ rise after RuR or tranilast treatment might be mediated by other mechanosensitive Ca^2+^-permeable channels, such as TRPC1, TRPV4, TRPM7 and TRPP2. Our data agreed with several previous reports, which showed TRPV2 being a hypotonocity-activated channel in other cell types, including murine aortic smooth muscle [25] and mouse odontoblasts [26].

Our findings may have significant pathological significance. HGPS cells displayed an increase in mechanosensitivity [9], [10]. Such increase in mechanosensitivity has been suggested to be a causative factor for necrotic and apoptotic vascular cell death, contributing to severe vascular diseases such as atherosclerosis [9]. However, the underlying mechanism of how an increase in mechanosensitivity could lead to cell death was not known. This present study provided a mechanistic explanation. From our results, it is conceivable that mechanical strain may stimulate TRPV2, which was excessively expressed in HGPS vascular cells and caused a sustained Ca^2+^ entry, leading to Ca^2+^ overload and subsequent vascular cell death. Indeed, our data clearly showed that TRPV2 mediated the hypotonicity-induced activation of caspase 8, an index for apoptosis, in iPSC-ECs from HGPS patients. These data were in agreement with the generally accepted notion that Ca^2+^ overload could result in cell death [17], [18]. Interestingly, TRPV2-mediated Ca^2+^ overloading was previously reported to contribute to muscular dystrophy and/or cardiomyopathy in humans and animal models [29].

In conclusion, we found that an upregulation in TRPV2 expression is responsible for the sustained [Ca^2+^]_i_ elevation under mechanical stress in iPSC-ECs from HGPS patients. This could be an underlying reason for mechanical stress-induced vascular cell death in HGPS patients.

## Methods

### Generation and culture of human iPSC-ECs

IMR90-iPSCs (from normal individual, passage 15 to 25, WiCell Research Institute, Madison, WI) and HGPS-iPSCs (from passage 30–34) were undifferentiated human iPSCs derived from normal individuals and HGPS patients, respectively [20]. The cells were maintained on Matrigel™ (BD Biosciences, MA)-coated dishes with mTeSR™ medium (Stem Cell Technologies, BC, Canada) [20]. Differentiation of endothelial-like cells from human iPSCs was performed as described [30]. Briefly, embryoid bodies (EBs) were generated by digesting the human iPSCs with 1 mg/ml dispase (Gibco, Gaithersburg, MD) and the cell clusters were then re-suspended in differentiation medium which consisted of knockout-DMEM with 20% fetal calf serum (Hyclone, Logan, UT), 2 mM L-glutamine, 0.1 mM non-essential amino acids and 0.1 mM β-mercaptoethanol (Invitrogen, Carlsbad, CA) in non-coated dishes for 9 days. The EBs were then plated on gelatin-coated dish for 7 days. Then the central portions of the attached EBs were manually dissected and isolated from the surrounding outgrowth cells for further expansion using endothelial growth medium-2 (EGM-2, Lonza, Walkersville, MD) for 14 days for endothelial-like cell differentiation. CD45^−^-CD31^+^ cells were then isolated by MoFlow XPD cell sorter (Beckman-Coulter, Fullerton, CA) and were ultimately designated as human iPSC-ECs. For characterization, human iPSC-ECs were treated with 4.8 µg/ml of DiI-labeled acetylated low-density lipoprotein (DiI-AcLDL, Molecular Probes, Eugene, OR) for 5 hours at 37°C. Cells were washed by phosphate buffered saline followed by fixation with 2% paraformaldehyde, and immunostained with 10 µg/ml of *Ulexeuropaeus* Lectin-FITC (Sigma Aldrich, St Louis, MO, USA) for 1 hr at room temperature [31]. Fluorescence-activated cell analysis (FACS) was performed with PE-labeled antibodies against CD31 (BD bioscience, San Jose, CA), vWF (Beckman Coulter, Indianapolis, IN) and kinase insert domain receptor (KDR; Sigma, St Louis, MO).

### Total RNA isolation and RT-PCR

Cells were incubated with 1 ml of Trizol reagent (Invitrogen, USA), and total RNA was extracted via following the manufacturer’s instructions. In brief, lyates were mixed with chloroform and centrifuged at 12,000 g for 15 min at 4°C. RNA was precipitated with isopropanol, and washed with 75% ethanol, followed by centrifugation at 7,500 g for 5 min at 4°C. The RNA pellet was air-dried, resuspended in RNase-free water. UV absorbance of the RNA samples was measured using a NanoDrop 3300 Fluorospectrometer (Thermo Fisher Scientific Inc, USA). The ratio of the absorbance at 260 and 280 nm was found to be 2.0, indicated its adequate purity. For cDNA synthesis, 1.5 µg of total RNA was mixed with reverse-transcription master mix using a high-capacity cDNA reverse transcription kits (AppliedBiosystems, USA). Reverse transcription reactions were performed in a thermal cycler (Bio-Rad Laboratories Inc, USA). Primer sequences are shown in table 1. cDNA samples (0.4 µl) and primers (0.5 µl) were added with 24.1 µl of AmpliTaqGold® DNA Polymerase (Applied Biosystems, USA). 35 cycles (94°C for 1 min, 58°C for 1 min, 72°C for 1 min) were performed with a PCR machine (PTC-200, MJ Research, USA). The amplified PCR products were run on a 1.5% agarose gels containing ethidium bromide.

**Table 1:** Primer sequences for PCR amplification of targets and endogenous genes.

Primer	Accession numbers	Primer sequence 5′-3′
TRPC1	NM_001251845.1	Forward 5′- CCAAGCCCCATGCAGTTGGCT-3′
		Reverse 5′- AGGTGGGCTTGCGTCGGTAAC-3
TRPC3	NM_001130698.1	Forward 5′-TGACTTCCGTTGTGCTCAAATATG-3′
		Reverse 5′-CCTTCTGAAGCCTTCTCCTTCTGC-3′
TRPC4	NM_016179.2	Forward 5′-TCTGCAAATATCTCTGGGAAGAATGC-3′
		Reverse 5′-AAGCTTTGTTCGTGCAAATTTCCATTC-3′
TRPC5	NM_012471.2	Forward 5′-GTGGAGTGTGTGTCTAGTTCAG-3′
		Reverse 5′-AGACAGCATGGGAAACAGGAAC-3′
TRPC6	NM_004621.5	Forward 5′-AGTCCGGCTTACCTGTCATTGT-3′
		Reverse 5′-ATGGAGAGAAGTTGCTGTTGGC-3′
TRPC7	NM_020389.2	Forward 5′-GGATGCAGATGTGGAATGGAAG-3′
		Reverse 5′-CGTCATTTTCTCTGTCCACCTG-3′
TRPV1	NM_080704.3	Forward 5′-ACGCTGATTGAAGACGGGAAGA-3′
		Reverse 5′-TGCTCTCCTGTGCGATCTTGTT-3′
TRPV2	NM_016113.4	Forward 5′-AGCAGTGGGATGTGGTAAGCTA-3′
		Reverse 5′-TTTGTTCAGGGGCTCCAAAACG-3′
TRPV3	NM_145068.2	Forward 5′-CGAGGATGATTTCCGACTGT-3′
		Reverse 5′-GGGTGCACTCTGCTTCTAGG-3′
TRPV4	NM_021625.4	Forward 5′-TGGGATCTTTCAGCACATCATC-3′
		Reverse 5′-GAGACCACGTTGATGTAGAAGG-3′
TRPV5	NM_019841.4	Forward 5′-GTGGACTTGCCCTTCATGTT-3′
		Reverse 5′-CACTTCCACATAGCGAAGCA-3′
TRPV6	NM_018646.2	Forward 5′-TTGAGCATGGAGCTGACATC-3′
		Reverse 5′-TCTGCATCAGGTGCTGAAAC-3′
TRPM1	NM_002420.4	Forward 5′-CAGTTCAATCACGGACCAGCAA-3′
		Reverse 5′-GTTAGGGACAAGCGAGGGATTC-3′
TRPM2	NM_003307.3	Forward 5′-CCAAACTGTCTGATGCTGGGAA-3′
		Reverse 5′-CGAGGATGAAGTGAGAGTGGTT-3′
TRPM3	NM_020952.4	Forward 5′-CAGAAAGTGAAGGTATGGCTGC-3′
		Reverse 5′-ACCCCAAAGCTCATCAGAACCA-3′
TRPM4	NM_017636.3	Forward 5′-TACAGGGCAACAGCGATCTCTA-3′
		Reverse 5′-CTTATGCACCGATTCCCACGTT-3′
TRPM5	NM_014555.3	Forward 5′-TTCCTGTTCATCGTGGGTGTCA-3′
		Reverse 5′-CAGTTCACACGGGCTTCATCAA-3′
TRPM6	NM_017662.4	Forward ’-GCACACAACGAAAAGCCCAACAGA-3′
		Reverse 5′-GTACAGGCACACCACATCTTTTCC-3′
TRPM7	NM_017672.4	Forward ’-GCACACAACGAAAAGCCCAACAGA-3′
		Reverse 5′-AGCCTCACATACCTTAGCTCTG-3′
TRPM8	NM_024080.4	Forward 5′-GATTTTCACCAATGACCGCCG-3′
		Reverse 5′-CCCCAGCAGCATTGATGTCG-3′
TRPA1	NM_007332.2	Forward 5′-GCTACTCTCTAAAGGTGCCCAAG-3′
		Reverse 5′-CGTTGTCTTCATCCATTACCAG-3′
TRPP1	NM_001009944.2	Forward 5′-ATGCCACGCTAGCACTGACG-3′
		Reverse 5′-CGTGTTGTTGACCTCCAGGC-3′
TRPP2	NM_000297.2	Forward 5′-TCCGATGATGCAGCTTCCCAGAT-3′
		Reverse 5′-AATGCCCCATTTTCCTTCACACTC-3′
β-actin	NM_001101.3	Forward 5′-ATGGATGATGATATCGCCGCG-3′
		Reverse 5′–CTCCATGTCGTCCCAGTTGGT-3′

### Immunoblots

Cells were rinsed with ice-cold PBS, harvested in lysis buffer containing protease inhibitor cocktail tablet (Roche Diagnostics GmbH, Germany), and centrifuged at 14,000xg for 20 minutes at 4°C. Equal amount of proteins were mixed with laemmi buffer, incubated at 70°C for 3 min. Protein samples were separated by using 12% SDS-polyacrylamide mini-gels. The gels were transferred to polyvinylidene-difluoride membrane using Trans-blot SD semi-dry electrophoretic transfer cell (Bio-Rad Laboratories, USA) and blocked with 5% skimmed milk in TBS buffer at room temperature for 2 hr. The membranes were then incubated with the primary antibodies against TRPV2 (VRL-1) (Novus Biologicals, CO, USA) or β-actin (Santa Cruz Biotechnology Inc., USA) overnight at 4°C. Immunoreactivity was detected with the secondary anti-rabbit or anti-mouse antibody conjugated to HRP (Santa Cruz Biotechnology Inc., USA). The blots were developed using a chemiluminescence's reagent (Bio-Rad Laboratories, USA), with films being exposed and analyzed using Image J (National Institutes of Health, USA).

### [Ca^2+^]_i_ measurement

The cells were loaded with 5 µM Fluo-4/AM or 10 µM Fura-2/AM in the presence of 0.02% pluronic F-12 for 1 hr, and maintained in normal physiological saline solution (NPSS). [Ca^2+^]_i_ measurement was performed in NPSS, isotonic solution, or hypotonic solution. NPSS contained in mM: 140 NaCl, 5 KCl, 1 MgCl_2_, 1 CaCl_2_, 10 glucose, 10 HEPES, pH 7.4. Isotonic solution contained in mM: 65 Na-aspartate, 5 KCl, 1 CaCl_2_, 1 MgCl_2_, 10 HEPES, 10 glucose, 140 mannitol, pH 7.4 with NaOH, ∼300 mOsm. Hypotonic solution contained in mM: 65 Na-aspartate, 5 KCl, 1 CaCl_2_, 1 MgCl_2_, 10 HEPES, 10 glucose, pH 7.4 with NaOH, ∼210 mOsm. Fluo-4 fluorescence was recorded and analyzed by FV1000 laser scanning confocal imaging system. [Ca^2+^]_i_ response was expressed as a ratio of real-time fluorescence (F_1_; excitation at 490 nm) relative to the intensity at the beginning of the experiment (F_0_), namely F_1_/F_0_. The peak magnitude of the transient phase of hypotonicity-induced [Ca^2+^]_i_ rise was measured from the onset of hypotonic stimulation to the peak of [Ca^2+^]_i_ rise. The magnitude of sustain phase of [Ca^2+^]_i_ rise was measured at 400 sec after hypotonicity treatment. Fura-2 dye was used to measure basal [Ca^2+^]_i_ level. The Ca^2+^-bound and -unbound Fura-2 fluorescence signals were measured by dual excitation wavelengths at 340 and 380 nm using InCyt Basic Fluorescence Imaging System (Intracellular Imaging, Cincinnati, OH). Resultant fluorescence signals were collected at 510 nm and was used to calculate [Ca^2+^]_i_ as described by Grynkiewicz *et al*. [32]. Calibration was done by measuring the fluorescence intensity of Fura-2 dye using commercial Ca^2+^ concentration standard with precisely known Ca^2+^ concentration (Molecular Probes, USA). R_min_ and R_max_ for Fura-2 were obtained by permeabilizing the cells with 10 µM ionomycin in the presence of 5 mM EGTA and 5 mM CaCl_2_, respectively [32], [33]. All experiments were performed at room temperature (∼23°C).

### Caspase-8 activity assay

The cells were seeded on 96-well plate (Corning, 3792) coated with Matrigel™ (BD Biosciences, MA) with mTeSR™ medium (Stem Cell Technologies, BC, Canada). Cells were grown for 48 hrs and then were incubated with hypotonic or isotonic solution in the presence or absence of tranilast (100 µM) for 10 minutes. Cells were then washed and incubated with culture medium for 1, 6, or 24 hr to determine the optimal time that was needed for apoptosis to develop, following by luminescent-based caspase 8 assay (Caspase-Glo 8, Promega, Maison, WI). We found that 24 hr was the optical time for caspase 8 activity to increase. Thus, all experiments were performed with 24 hr incubation.

### Statistical analysis

Data were expressed as mean ± SE. Statistics was performed using Prisms 4.0 software (GraphPad Software Inc., La Jolla, CA, USA). Statistical significances were determined by the unpaired student *t*-test and differences were considered significant at *P<0.05.
